# The diagnostic value of abdominal ultrasound in the progression of necrotizing enterocolitis in low-birth-weight neonates

**DOI:** 10.1097/MD.0000000000045427

**Published:** 2025-10-24

**Authors:** Lili Jia, Zhanli Li, Honghong Zhu, Xiaoya Ye, Shuyue Luo, Qianfei Wang

**Affiliations:** a Department of Neonatology, Xi’an People’s Hospital (Xi’an Fourth Hospital), Xi’an, Shaanxi, China; b Department of Nephrology, The First Affiliated Hospital of Xiamen University, Xiamen, Fujian, China.

**Keywords:** abdominal ultrasound, diagnostic value, intestinal wall activity, low-birth-weight neonates, necrotizing enterocolitis

## Abstract

The present study aims to assess the value of the intestinal wall thickness, intestinal peristalsis combined with intestinal wall blood flow signal monitored by abdominal ultrasound (AUS) in the diagnosis of necrotizing enterocolitis (NEC) in low-birth-weight infants. Low-birth-weight (<2500 g) infants with Bell I (suspected NEC) and Bell II (definite NEC) were enrolled, and the entire cohort of 130 infants was divided into Bell I (n = 84) group and Bell II (n = 46) group. The general clinical characteristics, the intestinal wall activity parameters, including intestinal wall thickness, intestinal peristalsis, combined with intestinal wall blood flow signal obtained from AUS, as well as intramural gas and portal venous gas monitored by AUS and abdominal X-ray, were reviewed. The multivariable logistic regression analysis and the area under the receiver operating characteristic curve (AUCs) were used to assess the value of the above parameters for diagnosing NEC. The receiver operating characteristic curve analysis showed significant differences (*P* = .015) in the AUCs for AUS and abdominal X-ray examinations. Logistic regression analysis identified that intramural gas (*P* =.005, odds ratio [OR]: 4.98), thinned bowel wall (<1.5 mm) (*P* =.004, OR: 7.081), reduced peristalsis (*P* = .001, OR: 7.405), reduced intestinal wall blood flow signal (*P* = .002, OR: 9.074) of AUS were independent diagnostic factors for definite NEC. Furthermore, the AUC for the logistic model of AUS was 0.839 (95% confidence interval: 0.764–0.913), which showed superior ability in diagnosing NEC. In conclusion, the AUS examination plays a crucial role in diagnosing NEC earlier in low-birth-weight infants. Intestinal wall thickness, intestinal peristalsis, and combined with intestinal wall blood flow signal showed good diagnostic ability for NEC.

## 1. Introduction

Necrotizing enterocolitis (NEC) is a common and severe gastrointestinal disease in neonates, which is also one important cause of serious neonatal complication and death for newborns.^[1,2]^ The mortality rate of children with NEC remains high, largely due to the failure to timely diagnose early complications. Moreover, as the survival rate of very low-birth-weight preterm infants has been increasing, the frequency of severe NEC in this group has also risen accordingly.^[2]^ It has been reported that the incidence of NEC in neonatal intensive care units is 2% to 5%, with a rate of 4.5% to 8.7% among very low-birth-weight infants and a mortality rate of 20% to 30%. The mortality rate among extremely low-birth-weight infants is even higher, ranging from 30% to 50.9%,^[3]^ suggesting a close relationship between birth weight and the incidence and mortality of NEC. Extremely low-birth-weight infants who survive NEC are at risk for severe neurodevelopmental disability and those with surgical NEC have a markedly increased risk of such delays (38% surgical NEC vs 24% medical NEC).^[4–6]^

The early symptoms of NEC are rather insidious. At first, the affected children usually only show nonspecific infection symptoms such as drowsiness, apnea, hypotension, and fluctuating body temperature. The digestive tract symptoms are not obvious, which can easily be mistaken for severe infection or sepsis, making early diagnosis difficult and delaying the condition. Early identification of the clinical manifestations of NEC, timely diagnosis and treatment are challenging but extremely important for reducing the mortality rate of NEC.^[7,8]^ Bell criteria help in the diagnosis of NEC. Bell criteria, commonly used for diagnosis, staging, and planning treatment of NEC, were divided into 3 stages (I, II, and III).^[4,9]^

For a long time, abdominal X-ray (AXR) examination has been one of the main methods for diagnosing NEC.^[10]^ However, at present, relying solely on AXR to diagnose early NEC, especially in extremely premature infants, has significant limitations. Abdominal ultrasound (AUS) examination is recommended for dynamic observation of changes in abdominal signs,^[3,11]^ which was valuable to diagnose early NEC. Due to its noninvasive, radiation-free and bedside dynamic monitoring features, AUS has gradually been applied in clinical practice.^[12–14]^ In 2017, the International Neonatal Consortium Conference proposed to include AUS as one of the important imaging diagnostic criteria for NEC.^[15]^

The diagnostic value of AUS in NEC is affected by body weight, but its diagnostic value in NEC of neonates with different body weights remains unclear. In the present study, we aimed to evaluate the diagnostic value of AUS in the progression of NEC in low-birth-weight neonates through determination of intestinal wall thickness, intestinal peristalsis and intestinal wall blood flow signal.

## 2. Materials and methods

### 2.1. Sample selection

This retrospective study was conducted from January 2021 and October 2024 at the Neonatology Department of Xi’an No. 4 Hospital, and approved by the Ethical Committee of the Xi’an No. 4 Hospital. The guardians of the included infants were informed of the study and signed the informed consent form. All procedures were carried out according to the Declaration of Helsinki.

Based on the NEC definition by Walsh and Kliegman,^[16]^ low-birth-weight (<2500 g) infants with Bell I (suspected NEC) and Bell II (definite NEC) were enrolled. Finally, we retrospectively analyzed 130 infants divided into Bell I (n = 84) group and Bell II (n = 46) group. All the infants underwent AUS and AXR examinations, and the time difference between the 2 examinations is <24 hours. The exclusion criteria were as follows: Diseases of all internal organs (such as infectious diseases, poisoning, malignant tumors, etc); Congenital malformations or developmental abnormalities of the intestinal tract; and Incomplete clinical data and diagnostic parameters.

### 2.2. Clinical data and diagnostic parameters

Maternal age, maternal hypertension, maternal diabetes, multiple births, gender, gestational age, birthweight, APGAR scores at 5 minutes, age at diagnosis of NEC, and breastfeeding were collected.

To minimize potential bias, all archived images of AUS and AXR in this study were reevaluated by 2 experienced ultrasound doctors and 2 experienced pediatric radiologists, independently, who were blinded to the clinical characteristics and NEC staging. For AUS examination, the information of intramural gas, portal venous gas, thickened bowel wall (>2.5 mm), thinned bowel wall (<1.5 mm), peristalsis and intestinal wall blood flow signal were collected. For AXR examination, the information of intramural gas and portal venous gas were collected.

### 2.3. Statistical analysis

Statistical analysis was performed using SPSS software (SPSS, Chicago). Inter-observer agreement was assessed using Cohen kappa coefficient (κ). All ambiguous cases were reviewed by a third senior expert to ensure diagnostic consensus. Categorical variables are demonstrated as percentages and were analyzed by the chi-square (*χ*^2^) test. Continuous variables with a normal distribution are described as mean ± standard deviation and were analyzed by 2-tailed unpaired Student *t* test. Multivariable logistic regression analysis was conducted to evaluate the independent factors for diagnosing NEC (odds ratio [OR], 95% confidence interval [CI]). The goodness-of-fit was evaluated using the Hosmer–Lemeshow method. The receiver operative characteristic (ROC) curve was applied to determine the area under the ROC curve (AUC). AUCs were used to evaluate the diagnostic performance by comparing the AUCs of each model. *P* value < .05 was considered significant.

## 3. Results

### 3.1. Clinical characteristics

**Figure 1.**
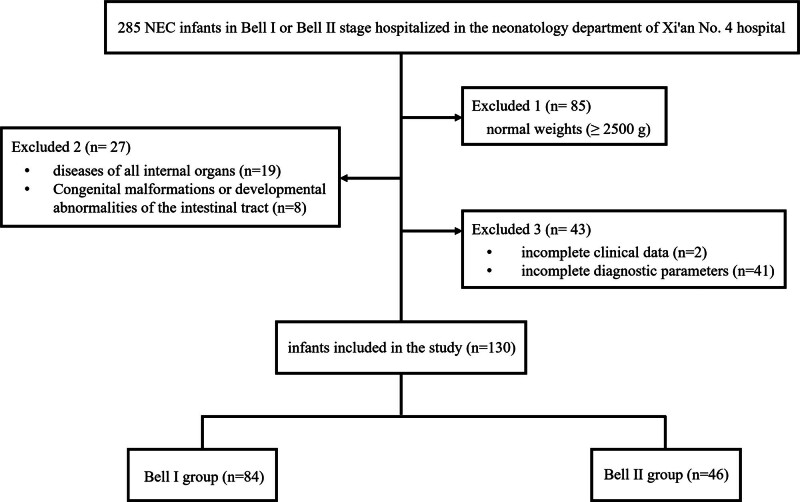
Flow chart of the study.

Out of 285 NEC infants in Bell I or Bell II stage hospitalized during the study period, 85 infants were excluded because of normal weights (≥2500 g). Twenty-seven infants were excluded because of diseases of all internal organs (n = 19) and congenital malformations or developmental abnormalities of the intestinal tract (n = 8). Forty-three patients were excluded for incomplete clinical data (n = 2) and incomplete diagnostic parameters (n = 41). Finally, a total of 130 NEC infants were enrolled in the study, including 84 infants in Bell I stage and 46 infants in Bell II stage (Fig. 1).

We analyzed the clinical characteristics for the infants with NEC and their maternal information, including maternal age, maternal hypertension, maternal diabetes, multiple births, gender, gestational age, birthweight, APGAR scores at 5 minutes, age at diagnosis of NEC, and breastfeeding. There were no significant differences between Bell I group and Bell II group regarding these data (*P* > .05, Table 1).

**Table 1:** Clinical characteristics between the 2 groups.

	Bell I group (n = 84)	Bell II group (n = 46)	*χ* ^2^	*P* value
Maternal age (yr), mean ± SD	29.7 ± 3.9	30.4 ± 4.3	0.86	.39
Maternal hypertension (%)	7 (8.3%)	5 (1.1%)	0.22	.63
Maternal diabetes (%)	17 (20.2%)	9 (19.6%)	0.08	.92
Multiple births (%)	11 (13.1%)	7 (15.2%)	0.11	.74
Male, n (%)	41 (48.8%)	29 (63%)	2.42	.12
Gestational age (wk), mean ± SD	34.8 ± 2.8	35.1 ± 2.5	0.67	.51
Birthweight (g), mean ± SD	1999.8 ± 479.5	2078.9 ± 44	0.92	.36
APGAR scores at 5 minutes, mean ± SD	9.1 ± 0.8	8.48.9 ± 0.7	0.33	.76
Age at diagnosis of NEC (d), mean ± SD	10.8 ± 3.6	11.2 ± 3.4	0.55	.58
Breastfeeding (%)	48 (57.1%)	22 (47.8%)	0.38	1.03

### 3.2. Description of AUS and AXR parameters

**Figure 2.**
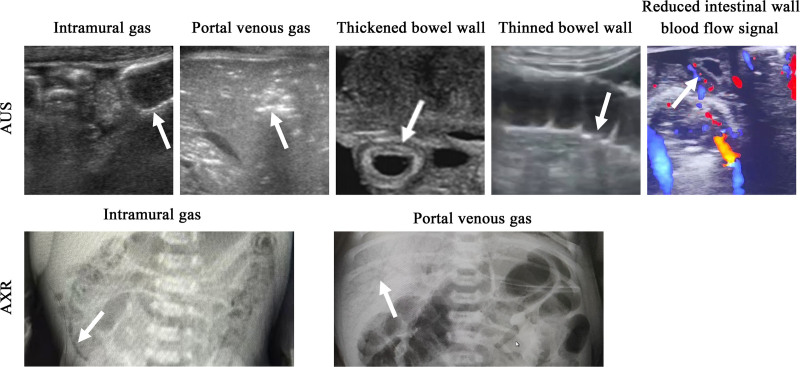
Representative AUS and AXR images of NEC. The white arrow indicates the target feature. AUS = abdominal ultrasound, AXR = abdominal X-ray, NEC = necrotizing enterocolitis.

Inter-observer agreement between 2 ultrasound doctors (κ = 0.78, *P* < .05) indicate excellent consistency. AUS examination revealed intramural gas (n = 28), portal venous gas (n = 13), thickened bowel wall (>2.5 mm, n = 13), thinned bowel wall (<1.5 mm, n = 8), reduced peristalsis (n = 15), reduced intestinal wall blood flow signal (n = 4) in Bell I group, while there were intramural gas (n = 29), portal venous gas (n = 15), thickened bowel wall (>2.5 mm, n = 14), thinned bowel wall (<1.5 mm, n = 17), reduced peristalsis (n = 15), and reduced intestinal wall blood flow signal (n = 4) in Bell II group. Furthermore, there were significant differences between Bell I group and Bell II group regarding these data (*P* < .05, Table 2). Inter-observer agreement between 2 radiologists (κ = 0.75, *P* < .05) indicate good consistency. For AXR examination, there were intramural gas (n = 12) and portal venous gas (n = 9) in Bell I group, while in Bell II group, there were intramural gas (n = 21) and portal venous gas (n = 12). Similarly, there were significant differences between Bell I group and Bell II group regarding these data (*P* < .05, Table 2). Representative AUS and AXR images of NEC were showed in Figure 2.

**Table 2:** Comparison of the AUS and AXR parameters of the 2 groups.

	Bell I group	Bell II group	OR	95% CI	*P* value
AUS, n (%)					
Intramural gas	28 (33.3%)	29 (63%)	3.412	1.610, 7.231	.001
Portal venous gas	13 (15.5%)	15 (32.6%)	2.643	1.125, 6.209	.023
Thickened bowel wall (>2.5 mm)	13 (15.5%)	14 (30.4%)	2.389	1.009, 5.661	.044
Thinned bowel wall (<1.5 mm)	8 (9.5%)	17 (37%)	5.569	2.169, 14.299	.001
Reduced peristalsis	15 (17.9%)	20 (43.5%)	3.538	1.579, 7.932	.002
Reduced intestinal wall blood flow signal	4 (4.8%)	15 (32.6%)	9.677	2.979, 31.441	.001
AXR, n (%)					
Intramural gas	12 (14.3%)	21 (45.7%)	4.971	2.139, 11.548	.001
Portal venous gas	9 (10.7%)	12 (26.1%)	2.941	1.132, 7.640	.023

Furthermore, in Bell I group, the detection rates for intramural gas (33.3%) and portal venous gas (15.5%) of AUS were both higher than those of AXR (14.3% and 10.7%). In Bell II group, the detection rates for intramural gas (63%) and portal venous gas (32.6%) of AUS were both higher than those of AXR (45.7% and 26.1%).

### 3.3. Diagnostic value of AUS and AXR parameters

**Figure 3.**
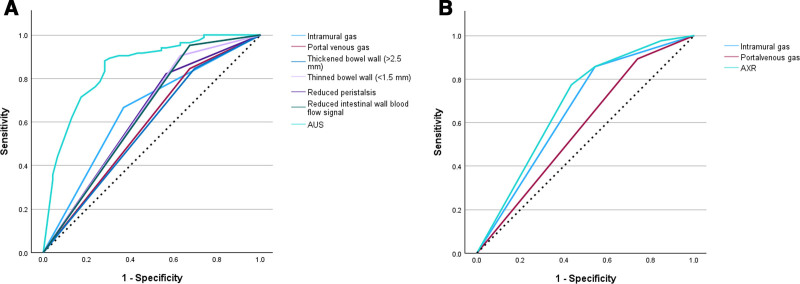
The ROC curves of AUS and AXR examinations for diagnosing NEC. AUS = abdominal ultrasound, AXR = abdominal X-ray, NEC = necrotizing enterocolitis, ROC = receiver operating characteristic.

The diagnostic value of AUS and AXR to diagnose NEC was measured in term of AUC. As shown in Figure 3, the AUC of AUS (0.893, 95% CI: 0.836–0.951) was significantly higher than the AUC of AXR (0.786, 95% CI: 0.711–0.862) (*P* = .015). Table 3 shows the predictive accuracies of the 2 examinations in diagnosing NEC.

**Table 3:** Diagnostic value of AUS and AXR.

	Sensitivity	Specificity	Accuracy	Positive-predictive value	Negative-predictive value
AUS	91.67%	86.96%	90.00%	92.77%	85.11%
AXR	84.52%	73.91%	80.77%	85.54%	72.34%

The performance of each AUS and AXR parameter to diagnose NEC was further assessed in term of AUC. For AUS examination, the AUC for intramural gas, portal venous gas, thickened bowel wall (>2.5 mm), thinned bowel wall (<1.5 mm), reduced peristalsis, reduced intestinal wall blood flow signal were 0.649 (95% CI: 0.549–0.748), 0.586 (95% CI: 0.480–0.691), 0.575 (95% CI: 0.469–0.680), 0.637 (95% CI: 0.532–0.742), 0.628 (95% CI: 0.524–0.732) and 0.639 (95% CI: 0.534–0.745), respectively (Fig. 3A). For AXR examination, the AUC for intramural gas and portal venous gas were 0.657 (95% CI: 0.554–0.760) and 0.577 (95% CI: 0.471–0.683) (Fig. 3B), respectively.

### 3.4. Multivariate logistic regression of AUS and AXR parameters

A multivariable logistic regression analysis was performed to assess AUS and AXR parameters. For AUS examination, we identified that intramural gas (*P* = .005, OR: 4.98), thinned bowel wall (<1.5 mm) (*P* = .004, OR: 7.081), reduced peristalsis (*P* = .001, OR: 7.405), reduced intestinal wall blood flow signal (*P* = .002, OR: 9.074) were independent diagnostic factors associated with NEC (Table 4). For AXR examination, we identified that intramural gas (*P* < .001, OR: 9.879) was an independent diagnostic factor associated with NEC (Table 4).

**Table 4:** Multivariate logistic regression of AUS and AXR parameters.

	Multivariate OR	95% CI	*P* value
AUS, n (%)			
Intramural gas	4.98	1.61, 15.409	.005
Portal venous gas	2.373	0.635, 8.87	.199
Thickened bowel wall (>2.5 mm)	1.977	0.582, 6.713	.274
Thinned bowel wall (<1.5 mm)	7.081	1.888, 26.559	.004
Reduced peristalsis	7.405	2.233, 24.551	.001
Reduced intestinal wall blood flow signal	9.074	2.24, 36.764	.002
AXR, n (%)			
Intramural gas	9.879	3.108, 31.401	<.001
Portal venous gas	1.524	0.451, 5.15	.498

**Figure 4.**
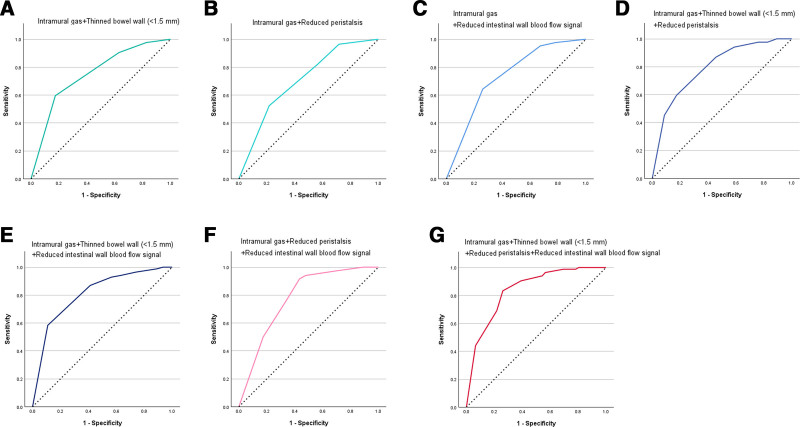
The ROC curves of different combinations of AUS parameters for diagnosing NEC. AUS = abdominal ultrasound, NEC = necrotizing enterocolitis, ROC = receiver operating characteristic.

Moreover, we built a logistic model to diagnose NEC according to the results of multivariable logistic regression analysis. We found the AUCs for intramural gas + thinned bowel wall (<1.5 mm), intramural gas + reduced peristalsis, intramural gas + reduced intestinal wall blood flow signal, intramural gas + thinned bowel wall (<1.5 mm) + reduced peristalsis, intramural gas + thinned bowel wall (<1.5 mm) + reduced intestinal wall blood flow signal, intramural gas + reduced peristalsis + reduced intestinal wall blood flow signal, and Intramural gas + thinned bowel wall (<1.5 mm) + reduced peristalsis + reduced intestinal wall blood flow signal were 0.75 (95% CI: 0.662–0.838) (Fig. 4A), 0.704 (95% CI: 0.609–0.800) (Fig. 4B), 0.733 (95% CI: 0.639–0.826) (Fig. 4C), 0.794 (95% CI: 0.712–0.875) (Fig. 4D), 0.811 (95% CI: 0.732–0.889) (Fig. 4E), 0.778 (95% CI: 0.688–0.868) (Fig. 4F) and 0.839 (95% CI: 0.764–0.913) (Fig. 4G), respectively.

## 4. Discussion

NEC is a severe disease in low-birth-weight infants, and it is generally diagnosed at Bell stage III, which increases the probability of surgery, postoperative complications and death.^[17,18]^ The mortality rate of infants with NEC requiring surgery is significantly higher than that of infants without the need for surgery (35% vs 21%).^[19]^ Besides surgical treatment, the mortality rate of NEC is also affected by gestational age, birth weight, and whether assisted ventilation was used on the day of NEC diagnosis.^[20,21]^ Therefore, for infants with low weight, early and accurate diagnosis to promptly initiate appropriate treatment is crucial for improving the prognosis in the later stage.

Clinical suspected diagnosis is usually confirmed by AXR, and Bell also used AXR to develop the famous NEC staging system. However, some studies have used AUS as an auxiliary measure for the diagnosis and treatment of infant NEC. This imaging method can detect typical NEC signs earlier and manage the disease more quickly.^[4]^ When comparing AUS with AXR in diagnosing NEC, studies have shown that AXR can depict intestinal wall thickness, intramural gas and portal venous gas to a certain extent, while AUS can also easily depict these conditions. Importantly, AUS can provide crucial additional information about intestinal wall viability.^[22]^ The significant value of AUS lies in its ability to directly depict some features of NEC that cannot be seen with AXR such as peristalsis and presence or absence of bowel wall perfusion. In addition, AUS has no ionizing radiation, which may be beneficial for the diagnosis and management of NEC.^[23]^

The production of intramural gas is due to the air in the intestine entry into the damaged intestinal wall, which is usually caused by gas-producing bacteria invading the damaged mucosa or the disruption of the mucosal barrier. Portal venous gas is originated from the absorption of intramural gas, entering the intestinal venous system and then the portal vein.^[24]^ Furthermore, AUS is possibly more sensitive, compared to AXR, to detect intramural gas, portal venous gas. In the present study, we found that whether in Bell I group or Bell II group, the detection rates of AUS for intramural gas and portal venous gas were both higher than those of AXR. For the parameters of intestinal wall viability, the detection rates of thickened bowel wall (>2.5 mm), thinned bowel wall (<1.5 mm), reduced peristalsis, reduced intestinal wall blood flow signal showed a significant elevation following the progression of NEC.

Subsequently, we explored the performance of AUS and AXR in diagnosing NEC from the perspective of AUC. In general, the AUC of AUS was significantly higher than the AUC of AXR. Multivariate logistic regression analysis was further conducted on the AUS and AXR parameters, and demonstrated that intramural gas, thinned bowel wall (<1.5 mm), reduced peristalsis, and reduced intestinal wall blood flow signal are independent diagnostic factors for NEC. Moreover, we built a logistic model to diagnose NEC according to the result of multivariable logistic regression analysis, and found that the AUC of logistic model was 0.839 (95% CI: 0.764–0.913), which was significantly superior than other different combinations of AUS parameters for diagnosing NEC.

In conclusion, we found that intramural gas, thinned bowel wall (<1.5 mm), reduced peristalsis, and reduced intestinal wall blood flow signal are independent diagnostic factors for NEC. The logistic model of AUS examination is crucial for diagnosing NEC in low-birth-weight infants. However, the study has several limitations. Firstly, this study was limited to the experience obtained by clinicians at a single center. Secondly, the sample size was not large enough. Further research with a larger sample size from more centers is needed to confirm the results of this study. Moreover, external validation in independent cohorts is necessary, and we are currently planning a prospective multi-center trial for this purpose.

## Author contributions

**Conceptualization:** Lili Jia, Zhanli Li.

**Data curation:** Lili Jia, Qianfei Wang.

**Formal analysis:** Lili Jia, Honghong Zhu, Shuyue Luo, Qianfei Wang.

**Funding acquisition:** Lili Jia.

**Methodology:** Lili Jia, Xiaoya Ye.

**Project administration:** Zhanli Li.

**Resources:** Honghong Zhu.

**Software:** Honghong Zhu.

**Supervision:** Xiaoya Ye.

**Validation:** Xiaoya Ye.

**Visualization:** Shuyue Luo.

**Writing – original draft:** Lili Jia.

**Writing – review & editing:** Zhanli Li.
